# Multiplex TaqMan qPCR assay for specific identification of encapsulated *Trichinella* species prevalent in North America

**DOI:** 10.1590/0074-02760180305

**Published:** 2018-10-29

**Authors:** Marcos de Almeida, Henry Bishop, Fernanda S Nascimento, Blaine Mathison, Richard S Bradbury, Alexandre da Silva

**Affiliations:** 1 Centers for Disease Control and Prevention, Division of Parasitic Diseases and Malaria, Center for Global Health, Atlanta, GA, USA; 2 US Food and Drug Administration Center for Food Safety and Applied Nutrition, Office of Applied Nutrition and Safety Assessment, Laurel, MD, USA

**Keywords:** *Trichinella* species, clinical/environmental infection, microscopy, real-time PCR, conventional PCR and sequencing, specific identification

## Abstract

BACKGROUND Human trichinellosis is a foodborne parasitic zoonotic disease caused by ingestion of raw or undercooked meat infected with nematode larvae of the genus *Trichinella*. In the USA, sporadic cases and outbreaks caused by the consumption of wild game meat infected with *Trichinella* have been reported. The current methods for diagnosis such as serology and microscopy are not specific, may result in false negative results, and cannot differentiate encapsulated *Trichinella* larvae to species level. The molecular protocols currently available for the differentiation of all encapsulate *Trichinella* species prevalent in North America have some limitations such as the inability to identify and resolve the presence of several *Trichinella* species in a single test. OBJECTIVES/METHODS In this study we developed and evaluated a multiplex TaqMan quantitative real-time polymerase chain reaction (qPCR) assay, which can simultaneously detect, identify and differentiate all species of encapsulated *Trichinella* occurring in North America i.e., *T. nativa*, *T*. *spiralis*, *T*. *murrelli* and *Trichinella* T6, even in cases of multiple infection in a single sample. We investigated two human biopsies and 35 wild animal meat samples considered as having a high likelihood of harboring *Trichinella* larvae obtained from the United States during 2009-2017. FINDINGS Using the multiplex assay describe here, 22 (59%) samples that tested positive contained *Trichinella* spp., were identified as: *T*. *nativa* (n = 7, including a human biopsy), *T*. *spiralis* (n = 9, including a human biopsy), *T*. *murrelli* (n = 3), *Trichinella* T6 (n = 1). Results also included two rare mixed infection cases in bears, a *T*. *nativa*/*T*. *spiralis* from Alaska and a *T*. *spiralis*/*Trichinella* T6 from California. The species identifications were confirmed using a conventional PCR targeting the rRNA ITS1-ITS2 region, followed by DNA sequencing analysis. The estimated limit of detection (LOD) was approximately seven larvae per gram of meat. MAIN CONCLUSIONS Differentiation of *Trichinella* spp. is needed to improve efforts on identification of case, optimize food safety control and better understand the geographic distribution of *Trichinella* species. The *Trichinella* qPCR multiplex proved to be a robust, easy to perform assay and is presented as an improved technique for identification of all known encapsulated species occurring in North America continent.

Trichinellosis is a foodborne parasitic zoonotic disease caused by nematodes of the genus *Trichinella*. The disease represents both a public health hazard and a food safety problem in many parts of the world.^1^ ^,^ ^2^ ^,^ ^3^

Human infection is acquired by ingestion of raw or undercooked infected meat of wild and game animals, and domestic (i.e., home-raised) or commercial pork meat. Although improvements in animal husbandry practices and implementation of federal programs to control the quality of processed meat products have contributed to the reduction in the number of reported cases of human trichinellosis since 1990’s in the United States, occasional outbreaks and sporadic cases associated with consumption of wild game meat continue to occur.^4^ ^,^ ^5^ ^,^ ^6^ ^,^ ^7^

Trichinellosis is a low prevalence disease in the United States and human infections may present with symptoms commonly observed in other diseases,^8^ contributing to delays in diagnosis of individual cases and identification of outbreaks. Standard laboratory methods used for diagnosis such as serology (the currently accepted gold standard diagnostic method) and microscopy may produce false negative results depending on the stage of disease and the larval load in the sample.^1^ ^,^ ^8^ In addition, using microscopy analysis, *Trichinella* species can only be differentiated to whether they are encapsulated or not, which does not allow precise identification of all species classified to date. As parasite capacity to survive freezing temperatures varies by species,^9^ ^,^ ^10^ the ability to differentiate species has important epidemiological and food surveillance implications. Therefore, efficient laboratory methods are needed to improve identification of clinical and environmental cases and better understand disease dissemination and the geographic distribution of the various *Trichinella* species in the North America.

Molecular procedures have the advantage of being both sensitive and specific for the identification of *Trichinella* parasites and have been applied to several epidemiological and taxonomic studies. As traditional testing approaches cannot distinguish *Trichinella* to species level, polymerase chain reaction (PCR)-based methods, which typically have higher sensitivity compared to microscopy, are useful for the identification of a variety of *Trichinella* species,^11^ ^,^ ^12^ including those causing human infection,^3^ ^,^ ^5^ ^,^ ^6^ ^,^ ^13^ and mixed infections in cases reported from focal areas of Europe and South Africa.^14^ ^,^ ^15^ ^,^ ^16^

Quantitative real-time PCR (qPCR) platforms are advantageous in comparison to conventional PCR tests, as they are more accurate, reduce the risk of contamination of the laboratory environment, and are less time consuming and labor intensive. The qPCR platforms have been increasingly applied to the detection and specific identification of *Trichinella* species.^17^ ^,^ ^18^ However, the protocols currently available cannot identify and resolve the presence of several *Trichinella* species in a single assay.

To facilitate the identification of clinical and environmental trichinellosis cases, we developed a multiplex TaqMan qPCR assay targeting the ribosomal RNA ITS1 region (rRNA-ITS1), for the specific identification of four encapsulated *Trichinella* species known to occur in North America. We analyzed meat samples implicated as the source of outbreaks or sporadic cases of human trichinellosis, comparing the multiplex TaqMan qPCR assay with microscopy results.

## MATERIALS AND METHODS

*Sample selection* - Two clinical samples and 35 wild game meat samples submitted to the CDC’s Parasitic Diseases Reference Diagnostic Laboratory between 2009-2017 were analyzed. The meat samples were obtained from animals hunted in several US states: 12 of walrus (Alaska), 11 samples of bear (Alaska n = 3, California n = 7, Wisconsin n = 1), five of boar (Minnesota n = 2, Missouri, California and Washington n = 1, respectively), four of deer (California n = 3 and Missouri n = 1), one of mountain lion (Idaho), one boar/deer mixed sausage (Missouri) and one deer/bear mixed sausage (California). All samples were considered to be potential sources for either outbreaks or individual cases of trichinellosis in humans. In addition, *T*. *nativa* (code ISS70 and ISS1751), *T*. *spiralis* (code ISS3 and ISS328), *T*. *murrelli* (code ISS346 and ISS415), *Trichinella* T6 (code ISS34 and ISS40) and *T*. *pseudospiralis* (code ISS13, ISS470 and ISS4134) larvae isolates were provided by Dr. Edoardo Pozio, Instituto Superiore di Sanita - European Union Reference Laboratory for Parasites for use as reference controls. Samples are listed in Table I.

*Microscopic analysis* - Initial analysis of the 37 samples was performed by compressing fragments of meat (roast and steak) of approximately 0.25 g in weight between two glass slides. These samples were examined by two independent expert analysts using light microscopy at 100x magnification to detect larvae of *Trichinella* spp. If no larvae were detected, a larger aliquot of the sample, determined by the total amount of the sample available, was partially digested using artificial gastric juice (aqueous solution of 0.5% pepsin and 0.7% hydrochloric acid), incubated at 37ºC for a minimum of 4 h, and examined.^19^ The microscopic analysis reproducibility was verified by reexamining a blinded panel of 12 new aliquots of these 37 samples. Larval loads for the determination of limit of detection (LOD) of the novel *Trichinella* multiplex qPCR were determined by digestion of approximately 0.25 g of three *Trichinella* positive meat samples, then counting the number of larvae within each sample.

*DNA extraction* - DNA was extracted from approximately 0.25 g cubes of meat and from an average of five larvae of each reference control isolates using DNeasy Blood & Tissue Kit (Qiagen, Germantown, MD), following the manufacturer’s instructions.

*Oligonucleotide design and TaqMan real-time PCR optimization* - Generic ribosomal RNA (rRNA) gene sequences of the different nematode species, including *Trichinella* spp. available in GenBank were aligned and used to design specific oligonucleotides to amplify fragments of approximately 3540 bp encompassing the full-length 18S-ITS1-5.8S-ITS2 rRNA of *T*. *nativa*, *T*. *murrelli*, *T*. *spiralis* and *Trichinella* T6. These DNA fragments were cloned using pCR2.1-TOPO vector (Invitrogen-Thermo Fisher, Waltham, MA) following the manufacturer directions and sequenced for reference (data not shown). The *Trichinella* multiplex qPCR assay was prepared using primers TCN-ITS1 2307F and TCN-ITS1 2411R, specific for the four known encapsulated *Trichinella* species occurring in North America. The primers were designed to amplify fragments of rRNA-ITS1 region ranging from 85 to 98 bp depending on the species (nucleotides 2285 to 2370, based on *T*. *nativa* accession no. KP307962). Four species-specific probes TnatR, TsprlR, TmurR and T6R labeled with VIC, CY5, FAM and NED and using minor grove binding (MGB) quenchers (Applied Biosystems-Thermo Fisher, Waltham, MA) were designed for differential detection of *T*. *nativa*, *T*. *spiralis*, T. *murrelli*, and *Trichinella* T6, respectively. Oligonucleotide sequences, nucleotide biding position (target) and GenBank references are listed in Table II.

**Fig. 1:**
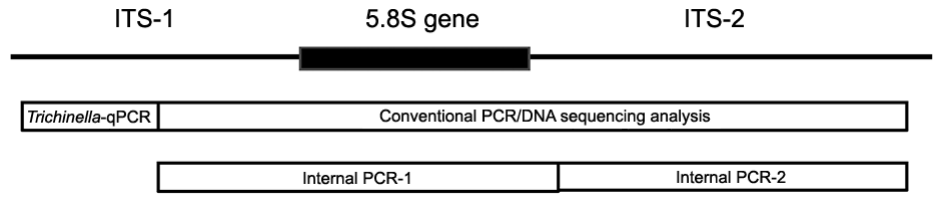
polymerase chain reaction (PCR) targets at rRNA-ITS region. *Trichinella* quantitative real-time (qPCR) corresponds to nucleotides 2285 (TCN-ITS1 2307F ) to 2370 (TCN-ITS1 2411R). Conventional PCR/DNA sequencing analysis corresponds to nucleotides 2375 (TCN-ITS1 2407F) to 3519 (TCN-28S 3511R). Internal PCR 1 corresponds to nucleotides 2375 (TCN-ITS1 2407F) to 3006 (TCN-ITS2 3020R) and Internal PCR 2 corresponds to nucleotides 3006 (TCN-ITS2 302F) to 3519 (TCN-28S 3511R). *T. nativa* accession no. KP307962 was used as a reference.

The specificity of the *Trichinella* multiplex qPCR assay was evaluated by comparing results with an in-house ITS1-ITS2 conventional PCR (Table II), followed by DNA sequencing analysis. These reactions were prepared with genus-level primers TCN-ITS1 2407F and TCN-28S 3511R designed to amplify fragments ranging between 1140-1167 bp depending on the species (nucleotides 2375-3519, *T*. *nativa* accession no. KP307962 as reference). In addition, internal primers TCN-ITS2 3020F TCN-ITS2 3020R were used for alternative amplification and for DNA sequencing analysis in cases where the amplification with ITS1-ITS2 PCR failed. Fig. 1 shows a diagram depicting the areas these PCR reactions targets within rRNA ITS1-5.8S-ITS2 sequence.

*SYBR Green methods used for quality control and comparison* - To assure the quality of the 37 DNA extracts and to minimize possible false negative results, a SYBR Green qPCR screening reaction were designed to amplify the animals DNA extracts using the generic primers 18SrRNA-F and 18SrRNA-R. In addition, another SYBR Green using the primers ESVF and ESVR,^20^ originally described to amplify *T*. *nativa*, *T*. *spiralis*, *T*. *britovi, T*. *pseudospiralis*, *T*. *nelsoni*, *Trichinella* T5 and *Trichinella* T6, were used to confirm the results on samples tested negative by the *Trichinella* spp. multiplex TaqMan qPCR.

**TABLE I:** List of samples, reference *Trichinella* spp. larvae isolates and nematode DNA tested by *Trichinella* quantitative real-time polymerase chain reaction (qPCR) multiplex assay and microscopy analysis. Species were identified using *Trichinella* qPCR multiplex assay and confirmed by DNA sequencing analysis of the *Trichinella* ribosomal RNA gene

Sample ID	Host, origin, year	*Trichinella* multiplex qPCR assay (Dye/Ct value)	*Trichinella* rRNA gene sequencing result	Microscopy result for 0.25 g of sample
SP#1	Human, MN, 2011	*T. spiralis* (CY5 - Ct28)	*T. spiralis*	NEG
SP#2	Human, AK, 2014	*T. nativa* (VIC - Ct28)	*T. nativa*	POS
SP#3	Bear, CA, 2006	*T. murelli* (FAM - Ct36)	*T. murelli*	2 larvae
SP#4	Bear, CA, 2007	*T. murelli* (FAM - Ct17)	*T. murelli*	POS
SP#5	Bear, CA, 2009	*T. murelli* (FAM - Ct25)	*T. murelli*	POS
SP#6	Boar, MN, 2011	*T. spiralis* (CY5 - Ct28)	*T. spiralis*	NEG
SP#7	Boar, MN, 2011	*T. spiralis* (CY5 - Ct26)	*T. spiralis*	NEG
SP#8	Boar, WA, 2012	NEG	NEG	NEG
SP#9	Bear, CA, 2012	NEG	NEG	NEG
SP#10	Bear, AK, 2013	*T. nativa* (VIC - Ct24) *T. spiralis* (CY5 - Ct32)	*T. nativa* *T. spiralis*	15 larvae
SP#11	Boar, MO, 2013	*T. spiralis* (CY5 - Ct30)	*T. spiralis*	POS
SP#12	Deer, MO, 2013	NEG	NEG	NEG
SP#13	Boar/Deer, MO, 2013	*T. spiralis* (CY5 - Ct37)	*T. spiralis*	NEG
SP#14	Mountain lion, ID, 2014	*Trichinella* T6 (NED - Ct30)	*Trichinella* T6	POS
SP#15	Bear, CA, 2015	*T. spiralis* (CY5 - Ct27)	*T. spiralis*	POS
SP#16	Bear, CA, 2015	*T. spiralis* (CY5 - Ct32)	*T. spiralis*	POS
SP#17	Bear, CA, 2015	*T. spiralis* (CY5 - Ct26)	*T. spiralis*	21 larvae
SP#18	Bear, AK, 2015	*T. nativa* (VIC - Ct25)	*T. nativa*	POS
SP#19	Bear, AK, 2016	NEG	NEG	NEG
SP#20	Bear, WI, 2016	*T. nativa* (VIC - Ct37)	*T. nativa*	POS
SP#21	Boar, CA, 2017	*T. spiralis* (CY5 - Ct26)	*T. spiralis*	POS
SP#22	Walrus, AK, 2017	*T. nativa* (VIC - Ct36)	*T. nativa*	NEG
SP#23	Walrus, AK, 2017	*T. nativa* (VIC - Ct35)	*T. nativa*	NEG
SP#24	Walrus, AK, 2017	NEG	NEG	NEG
SP#25	Walrus, AK, 2017	NEG	NEG	NEG
SP#26	Walrus, AK, 2017	*T. nativa* (VIC - Ct26)	*T. nativa*	NEG
SP#27	Walrus, AK, 2017	NEG	NEG	NEG
SP#28	Walrus, AK, 2017	*T. nativa* (VIC - Ct26)	*T. nativa*	POS
SP#29	Walrus, AK, 2017	NEG	NEG	NEG
SP#30	Walrus, AK, 2017	NEG	NEG	NEG
SP#31	Walrus, AK, 2017	NEG	NEG	NEG
SP#32	Walrus, AK, 2017	NEG	NEG	NEG
SP#33	Walrus, AK, 2017	NEG	NEG	NEG
SP#34	Dear, CA, 2017	NEG	NEG	NEG
SP#35	Dear, CA, 2017	NEG	NEG	NEG
SP#36	Dear, CA, 2017	NEG	NEG	NEG
SP#37	Dear/Bear, CA, 2017	*T. spiralis* (CY5 - Ct37) *Trichinella* T6 (NED - Ct25)	*T. spiralis* *Trichinella* T6	POS
*T. nativa* ^*a*^	Wolf, Russia, 1987 (ISS70) Bear, Canada, 2005 (ISS1751)	VIC positive - (Ct24)	*T. nativa*	NA
*T. spiralis* ^*a*^	Pig, Poland, 1960 (ISS3) Pig, Sweden, 1994 (ISS328)	CY5 positive - (Ct23)	*T. spiralis*	NA
*T. murrelli* ^*a*^	Bear, USA, 1994 (ISS348) Raccoon, USA, 1989 (ISS415)	FAM positive - (Ct25)	*T. murrelli*	NA
*Trichinella T6* ^*a*^	Grizzly, USA, 1983 (ISS34) Mountain lion, USA, 1985 (ISS40)	NED positive - (Ct28)	*Trichinella T6*	NA
*T. pseudospiralis* ^*a*^	Raccoon, Russia, 1972 (ISS13) Black vulture, USA, 1995 (ISS470) Boar, Italy, (ISS4134)	NEG	NA	NA
*Human/Helminths* ^*b*^		NEG	NA	NA

*a*: larvae isolates positive controls - Instituto Superiore di Sanita, European Union Reference Laboratory for Parasites; *b*: *Taenia solium*, *Paragonimus mexicanus*, *Trichuris* sp., *Halicephalobus* sp., *Anisakis simplex*, *Brugia malayi*, *Dirofilaria immitis*, *Onchocerca lupi*, *Onchocerca volvulus*, and *Wuchereria bancrofti*; NA: not available; NEG: negative; POS: positive.

**TABLE II:** Oligonucleotide sequences used for detection and identification of *Trichinella nativa*, *T*. *spiralis*, *T*. *murrelli* and *Trichinella* T6

Name	Primer sequences^*a*^	Type of oligo - Reaction	nt Target^*b*^	Reference^*c*^
TCN-ITS1 2307F	GAG TGT GAC CAA AAT GAG AAA CC	Primer - TaqMan	2285-2307 (ITS1)	KP307962
TCN-ITS1 2411R	CAA ACC TAT TGA AAC CCA AGC AC	Primer - TaqMan	2357-2370 (ITS1)	KP307962
TnatR	VIC-AAC ACA AAA AAT AAA C-MGBNFQ	Probe - TaqMan	2527-2342 (ITS1)	KP307962
TsprlR	FAM-AGC ACA TTA CAC TGC ACT-MGBFNQ	Probe - TaqMan	2338-2355 (ITS1)	KC006422
TmurR	FAM-AAC ACA CTG AGC ACT ACA-MGBNFQ	Probe - TaqMan	2335-2352 (ITS1)	KC006408
TT6R	NED-CAA ACA CTA AAA TAA-MGBNFQ	Probe - TaqMan	2322-2336 (ITS1)	KP307967
TCN-ITS1 2407F	GGT CAA CCG CCA CGT CCA ATC	Primer - PCR, sequencing	2375-2395 (ITS1)	KP307962
TCN-28S 3511R	CTC GCC GCT ACT TGG AGA ATT CG	Primer - PCR, sequencing	2497-3519 (ITS2)	KP307962
TCN-ITS2 3020F	TGT CGA CGT TGC AGT GTG TG	Primer - PCR, sequencing	2957-2976 (ITS2)	KP307962
TCN-ITS2 3020R	CAC ACA CTG CAA CGT CGA CA	Primer - PCR, sequencing	2957-2976 (ITS2)	KP307962
18SrRNA-F	TAT GCG ACT ACC ATG GTG ATA AC	Primer - SYBR Green qPCR	349-371 (18S)	NA
18SrRNA-R	CTG CCT TCC TTG GAT GTG GTA	Primer - SYBR Green qPCR	423-443 (18S)	NA
ESVF	GTT CCA TGT GAA CAG CAG T	Primer - SYBR Green qPCR	1srDNA	^(20)^
ESVR	CGA AAA CAT ACG ACA ACT GC	Primer - SYBR Green qPCR	1srDNA	^(20)^

*a*: primers are in 5’-3 ‘orientation; *b*: nucleotide binding position; *c*: GenBank accession references.

The optimal annealing temperatures for qPCR primers and probes were determined using a 2ºC gradient ranging from 56-64ºC. The optimal probe concentration was determined using a 50 nM gradient ranging from 50-500 nM. Two independent reaction sets of each gradient were prepared in triplicate to assure the quality of results. The annealing temperature of the conventional PCR was empirically determined based on primer nucleotide sequences.

The specificity of the multiplex qPCR assay was determined by comparison to 20 human and multiple helminth DNA extracts identified in our laboratory in other investigations (these being those of *Taenia solium*, *Paragonimus mexicanus*, *Trichuris* sp., *Halicephalobus gingivalis*. *Anisakis simplex*, *Brugia malayi*, *Dirofilaria immitis*, *Onchocerca lupi*, *Onchocerca volvulus* and *Wuchereria bancrofti*).

The LOD of the *Trichinella* qPCR assay was demonstrated on the basis of the number of larvae (larvae per gram, lpg) determined by microscopy in three of the samples meat (SP#3, SP#10 and SP#17). DNA was extracted from the 0.25 g of meat portions using the protocol described above and used in *Trichinella* qPCR reactions were prepared using a 10-fold DNA dilution gradient ranging from 10^-1^ to 10^-6^. LOD was calculated based on results of a triplicate set of reactions. In addition, another set of reactions prepared using dilutions of DNA extracts from larvae reference controls, were used to determinate a second parameter of LOD (data not shown).

*qPCR reactions* - The TaqMan reaction mixtures were prepared with 1-3 µL of DNA, 250 nM each primer, 150 nM of each probe, 12.5 µL of TaqMan® Universal PCR Master Mix (Applied Biosystems - Thermo Fisher, Waltham, MA) and sterile water to adjust the volume to 25 µL. The reactions were performed on an ABI 7500 real-time PCR system (Applied Biosystems - Thermo Fisher, Waltham, MA) using the following cycling parameters: 50ºC for 2 min, 95ºC for 10 min, followed by 40 cycles of 95ºC for 15 s and 60ºC for 1 min. Fluorescence data were collected at the end of each 60ºC plateau.

The reaction mixtures for the two SYBR Green assays were prepared with 3 µL of DNA, 250 nM of each primer, 12.5 µL of QuantiTect SYBR Green PCR master mix (Qiagen, Germantown, MD) and sterile water to adjust the volume to 25 µL. The assays were run side-by-side on ABI 7500 real-time PCR system (Applied Biosystems - Thermo Fisher, Waltham, MA) using the following cycling parameters: 50ºC for 2 min, 95ºC for 15 min, followed by 40 cycles of 95ºC for 15 s and 60ºC for 1 min. Fluorescence data were collected at the end of each 60ºC plateau.

*Conventional PCR and DNA sequencing analysis* - Reactions were prepared with 3 µL of DNA, 250 nM of each primer and 45 µL of Platinum Blue PCR SuperMix mixture (Invitrogen - Thermo Fisher, Waltham, MA) in a total volume of 50 µL. PCR was performed in a GeneAmp PCR System 9700 thermocycler (Applied Biosystems - Thermo Fisher, Waltham, MA). Reactions were performed using the following cycle structure: 95ºC for 2 min, followed by 40 cycles of 95ºC for 30 s, 60ºC for 30 s, and 72ºC for 1 min, with a final extension of 72ºC for 5 min. The amplicons were resolved in a 1.5% agarose gel, purified with StrataPrep PCR Purification Kit (Stratagene, San Diego, CA) and sequenced using BigDye version 3.1 chemistry (Applied Biosystems - Thermo Fisher, Waltham, MA) with primers TCN-ITS2 3020F and TCN-ITS2 3020R in addition to those used for PCR amplification (TCN-ITS1 2407F and TCN-28S 3511R). The sequencing reaction mixtures were purified through Sephadex Multi-Screen-HV plates (Millipore) and analyzed on an ABI Prism 3100 sequencer, with data collection software, version 2.0, and DNA Sequencing Analysis Software, version 5.1 (Applied Biosystems - Thermo Fisher, Waltham, MA). The sequences were assembled, edited, and aligned in DNA STAR SeqMan v. 14.0.0 (88) 422 (DNASTAR Inc., Madison, WI) software.

For specific identification of parasites in the specimens containing more than one species, DNA fragments amplified using primers TCN-ITS1 2407F/ TCN-ITS2 3020R and TCN-ITS2 3020F/ TCN-28S 3511R were used to prepare libraries using ThruPlex-FD Prep Kit (Rubicon Genomics, Ann Arbor, MI).

Amplicon library were prepared using 20 ng of input DNA and 10 cycles of amplification; purification was performed as per manufacturer’s instructions. The libraries were quantified using a Quibit fluorometer 2.0 with HS reagent (Invitrogen -Thermo Fisher, Waltham, MA), NEBNext Library Quantification kit for Illumina (New England Biolabs, Ipswich, MA) and the library size was assessed using Tape Station 2200 gDNA Screen Tape (Agilent, Santa Clara, CA). The barcoded libraries were diluted to 4 nM pooled and, 15 pM loaded in a MiSeq Nano Kit 2x250 (Illumina, San Diego, CA). MiSeq reads were analyzed using MAFFT multiple alignment available in Geneious V.9 GeniousR9 - (9.1.5) software.

*Ethics* - Clinical samples were used in accordance with a CDC human subject-approved protocol - IRB# 5756.

## RESULTS

*Reproducibility of microscopy analysis* - Encysted *Trichinella* larvae were identified in 15 of the 37 samples (40.5%) by microscopic analysis, including one of the two human biopsy specimens (Table I). New aliquots were prepared from 12 of the 37 samples for the reproducibility exercise (i.e., SP#1, SP#3 SP#7, SP#8, SP#9, SP#10, SP#11, SP#13, SP#14, SP#16, SP#17 and SP#29). Eight samples that were positive on first examination were also positive when re-examined. Samples SP#11 and SP#14, which were positive upon the first examination by microscopy, were negative when re-examined; Samples SP#8 and SP#9 were negative by both examinations.

**Fig. 2:**
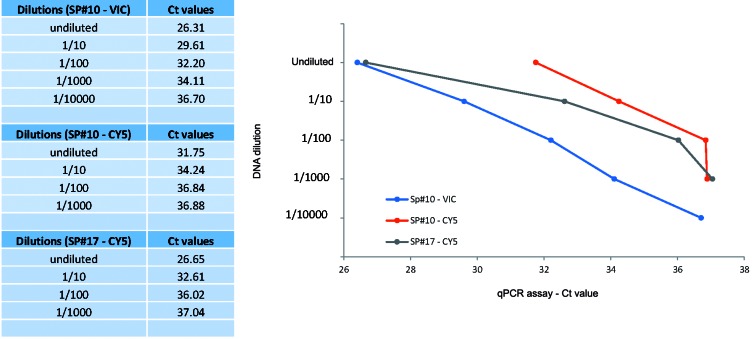
*Trichinella* multiplex quantitative real-time polymerase chain reaction (qPCR) assay limit of detection (LOD). SP#10 (*T. spiralis* and *T. nativa* mixed infection) and SP#17 (*T. spiralis* only) were tested in triplicates of 10-fold series dilutions. The Ct values on the plots are the average of three runs of each sample.

Larval loads in samples SP#3, SP#10 and SP#17 were determined as 8, 60 and 80 larvae/g (lpg) of meat, respectively (Fig. 2). DNA extracts from these naturally infected samples were used for the demonstration of the *Trichinella* qPCR multiplex assay LOD as described below.

*Quality control, optimization and evaluation of LOD of Multiplex TaqMan qPCR reactions* - All 10 *Trichinella* larvae DNA control extracts diluted at 1/50 were successfully amplified using this assay. The rRNA gene full-length used as reference sequences on oligonucleotides design were submitted to GenBank database under accession# KC006408-KC006421 (*T*. *murrelli*), KC006422-KC006433 (*T*. *spiralis*), KP307962-KP307966 (*T*. *nativa*) and KP307967-KP307971 (*Trichinella* T6). The amplification of all 37 DNA extracts using the generic 18SrRNA SYBR Green qPCR demonstrated the integrity and absence of inhibitors in these DNA samples.

**Fig. 3:**
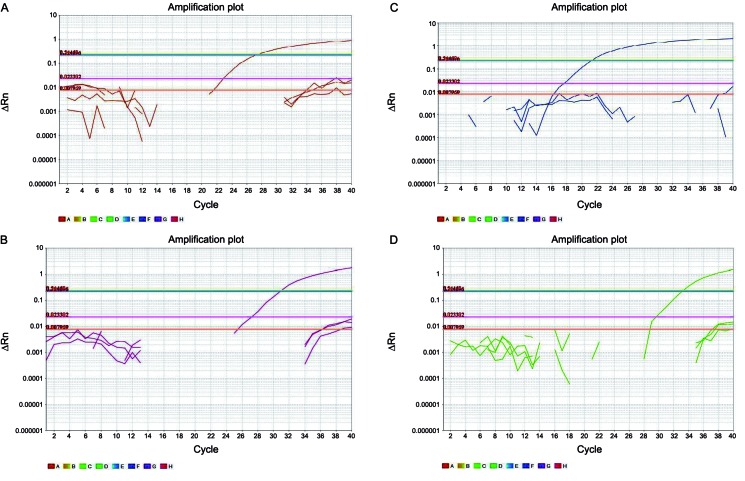
amplification plots of *Trichinella* quantitative real-time polymerase chain reaction (qPCR) multiplex assay using *T. nativa*, *T. spiralis*, *T. murrelli* and *Trichinella* T6 specific probes. Threshold baselines are colored in blue - VIC -*T. native* (panel A), yellow - FAM - *T. murrelli* (panel B), violet - CY5 - *T. spiralis* (panel C) and green - NED - *Trichinella* T6 (panel D).

The optimal *Trichinella* multiplex qPCR assay annealing temperature and probe concentration were determined by gradient analysis to be between 58-62ºC with between 100 to 200nM of probe per reaction. Based on these results, the optimal qPCR conditions were determined to be an annealing temperature of 60ºC and 150nM of probe per reaction. The capacity of the assay to correctly identify and differentiate *Trichinella* species was determined using a combination of all probes in individual reactions containing *T*. *nativa*, *T*. *spiralis*, *T*. *murrelli*, *Trichinella* T6 and *T. pseudospiralis* DNA controls. With exception of *T. pseudospiralis*, we were able to detect and identify the species investigated (Table I, Fig. 3). DNA aliquots from human and the other helminth species were not amplified by the qPCR assay.

Using DNA extracts of samples SP#3, SP#10 and SP#17, the LOD calculated for the *Trichinella* multiplex qPCR assay was 7x10^-3^ lpg of infected meat. *T*. *murrelli* was identified in an undiluted DNA aliquot of SP#3 (FAM - Ct 37). In SP#17, *T*. *spiralis* was detected in an undiluted aliquot (Ct 26) and in 1/10 (Ct 30), 1/100 (Ct 33) and 1/1000 (Ct 37) dilutions. A mixed infection with both *T*. *nativa* and *T*. *spiralis* was detected in SP#10, *T*. *nativa* was detected in an undiluted aliquot (Ct 24), and in 1/10 (Ct 28), 1/100 (Ct 35) and 1/1000 (Ct 37) dilutions and *T. spiralis* was detected in an undiluted DNA aliquot only (Ct 37). We also ran *Trichinella* multiplex qPCR gradient reactions using *T*. *nativa*, *T*. *spiralis*, *T*. *murrelli*, *Trichinella* T6 DNA controls, these PCR preparations showed reactivity with DNA dilutions equivalent to < 10^-15^ g/µL (data not shown).

*Trichinella spp. identification in clinical and meat samples.* The qPCR multiplex assay detected *Trichinella* spp. in 22 out of 37 samples (59%, which included two human biopsies) with a total of 15 negative samples. The assay showed improved detection efficiency compared to conventional microscopic analysis since the 22 *Trichinella* spp. positive samples included the 15 samples positive by microscopy and seven other samples (SP#1, SP#6, SP#7, SP#13, SP#22, SP#23 and SP#26) that were negative by microscopic analysis. Results on the 22 cases infected with *Trichinella* spp. and the 15 negative samples were confirmed in samples investigated by the ESV/ESVR SYBR Green qPCR reactions.

Twenty singly infected samples were identified as; *T*. *nativa* (n = 7, including one clinical sample), *T*. *spiralis* (n = 9, including one clinical sample), *T*. *murrelli* (n = 3) and *Trichinella T6* (n = 1). The method also efficiently identified two cases with mixed infection. Meat sample SP#10 (bear - AK) contained a mixed infection of *T*. *nativa* (Ct 24)/*T*. *spiralis* (Ct 32) and sausage sample SP#37 (dear/bear mixed sausage - CA) was determined to be mixed infected with *Trichinella* T6 (Ct 24)/*T*. *spiralis* (Ct 37), representing a rare case of *T*. *spiralis* infection in Alaska and the first record indicating the presence of *Trichinella* T6 from California.

DNA sequencing analysis confirmed all single species infections identified by the *Trichinella* qPCR multiplex assay (including the two human biopsies). Species of the two mixed infection were confirmed by deep sequencing analysis. In both instances, amplicons of approximately 605bp and 490bp amplified using primers TCN-ITS1 2407F and ITS2 3020R or TCN-ITS2 3020F and 28S 3511R, respectively were used for definitive species identification. These results are summarized in Table I.

## DISCUSSION

The *Trichinella* qPCR multiplex assay described here was designed to target the rRNA coding region, since comparative DNA analysis performed in this study using ITS1-ITS2 region sequences revealed a significant difference among *T*. *spiralis* and *T*. *nativa*, *T*. *murrelli* and *Trichinella* T6. In agreement with our findings, other studies based on rRNA phylogenetic analysis of the genus *Trichinella*, showed similar levels of difference between these species.^12^ Compared to microscopy analysis, the assay was more sensitive for detection of clinical cases and proved to be very specific for species level differentiation of encapsulated *Trichinella* species found in North America.

Using our qPCR multiplex assay, 20 single *Trichinella*-infected and two *Trichinella*-mixed infected cases, i.e., *T*. *nativa*/*T*. *spiralis* and *T*. *spiralis*/*Trichinella* T6, were detected. The *T*. *spiralis*/*Trichinella* T6 mixed-infection was detected in a sausage sample (SP#37) which was prepared with a mixture of deer and bear meat. Since deer are atypical hosts for *Trichinella*,^7^ and we could not identify the two types of meat in the sausage, we presumed that the infection of this sample was from the bear meat.

Specific identification of *Trichinella* parasites does not necessarily affect patient treatment, but it may be important as the various species have a wide range of incubation periods and clinical manifestations of the disease seem to vary by infecting species.^5^ ^,^ ^21^ Although *T. spiralis* is the most common species implicated in human infection, recently identified human cases involving *T. murrelli* and *T. nativa* (inclusive in this study) exemplify the complexity of the epidemiology of trichinellosis and the potential for several species of this genus to cause infections in humans.^22^ ^,^ ^23^

The assay also allowed the identification of the first confirmed case of *Trichinella* T6 in a bear sample from the state of California and a rare case of *T*. *spiralis* in Alaska. *T*. *nativa* and *Trichinella* T6 seem to be confined to the cold climates areas of North America, including Alaska, Colorado, Idaho, Montana, Pennsylvania and Wyoming, due their tolerance to freezing temperatures. In contrast, *T*. *spiralis*, a species relatively intolerant to freezing temperatures, seems to be restricted to southern regions.^9^ ^,^ ^10^ However, environmental adaptation of larvae is an important mechanism for dissemination of *Trichinella* spp., which can explain the presence of *Trichinella* T6 and *T*. *spiralis* in regions with adverse temperatures.^9^ ^,^ ^10^ Finally, improvements in techniques for the isolation and detection of these parasites may also explain some of the apparent expansion of *Trichinella* spp. into newly reported regions.^4^ Use of sensitive and specific detection methods that allow the identification of single and mixed infections is important for epidemiological studies of the geographical distribution of species in the United States.

The detection and identification of *Trichinella* larvae in meat samples can greatly be influenced by the larval load and by the method used for this purpose.^1^ ^,^ ^24^ The discrepant results observed between molecular and microscopy analysis may be partially attributable to sampling effect, as larvae are not uniformly distributed throughout a meat sample. Supporting this theory, it should be noted that 2/12 (16%) of samples reexamined by microscopy were found to be negative on repeat microscopic analysis. This result reflects the importance of pooling different portions of the meat before performing the microscopy or PCR analysis for a conclusive diagnosis of the suspect cases. However, given the rate of detection of larval DNA in negative meat samples by microscopy, this study also highlights the relative insensitivity and lack of reproducibility of microscopy for the detection of *Trichinella* spp., meanly in cases when the parasite load is low.

The novel qPCR assay does not detect *T. pseudospiralis*, which is known to be present in North America, and has been reported from several animals, including cougars (*Puma concolor cougar*),^25^ Florida panthers (*Puma concolor coryi*)^26^ and wild boar.^27^ To date, while no human cases associated with this species have occurred on this continent, both sporadic cases of infection and outbreaks of trichinellosis due to *T. pseudospiralis* have occurred elsewhere.^28^ ^,^ ^29^ Unlike other *Trichinella* species in North America, the differentiation of this species from other trichinellae by microscopy is possible, as the larva is not encapsulated. However, in cases where infection with *T. pseudospiralis* is suspected and no larvae are found by microscopy, the use of *T. pseudospiralis* specific qPCR is advisable.^30^

The multiplex qPCR assay proposed here represents an advance in *Trichinella* spp. identification, and has an important impact on clinical cases. To our knowledge, there are no other methods published that offer the same level of specificity in a single, easy to perform assay for the identification of all encapsulated *Trichinella* species occurring in North America. Other protocols, designed for multi-species identification, rely on the use of multiple primers/probes, require multiple specific reactions to distinguish species, or are designed for single species identification.^17^ ^,^ ^18^

In conclusion, the molecular assay presented in this study offers improved detection and specific identification of the four encapsulated *Trichinella* species occurring in North America, including rare mixed infected cases. The data generated by such an assay has immediate implications for clinical epidemiological investigations and food safety, informing likely food sources for cases and indicating susceptibility of the larval *Trichinella* to freezing based on the species determined.
